# Diabetes

**Published:** 1895-05-11

**Authors:** 


### DIABETES.

(*Continued from page* 385, *Vol. XVII.*)

**Pathology.**—It was to be expected that Pavy's lectures would rouse much criticism. Noel Paton denies his proofs that (1) there is no increase of sugar in the blood leaving the liver, that (2) glycogen is not converted into sugar, and that (3) the latter is all excreted in the urine without any disappearing in the circulation otherwise. Paton[29] points out that phenyl hydrazine does not only yield crystals with carbohydrates, hence it affords no test of the derivation of them from proteids. Again, that the blood of the right heart is largely from other areas besides the liver, so that it cannot be a measure of the sugar produced in the latter; and on the other hand Bock and Menkowski have by excluding the liver from the circulation rendered the blood free of sugar. Pavy's argument that glycogen is converted into sugar when the liver cells are destroyed does not prove that they themselves are not the agents during life, of which Paton gives evidence. For no one denies that an amylolyth ferment may also be developed post-mortem. Baish's experiments show that healthy urine contains only a fourth or less of the amount which Pavy finds in it and in the blood. Hence there is a surplus used up in the blood. The intestinal cells, which he found to contain fat after a meal of oats, might well do so, for oats contain 5 per cent of fat. There is, therefore, no proof of the conversion of carbo-hydrates into fat, and, finally, since proteids can give off ·2 per cent. of their substance as carbo-hydrates, there should be only ·2 parts of sugar to the 35 of urea given off by 100 parts of proteids, but the sugar ratio to urea is often far higher than this even on a strict diet.

Hauseman[30] holds that diabetes may be of pancreatic origin or otherwise. Atrophy is the special type of lesion found in the pancreas when that organ is the cause of the disease. Williamson[31] has investigated microscopically the state of the pancreas in fifteen cases of diabetes. In only six was there any lesion likely to produce the disease, and in five cases with wasting the pancreas was normal. Hence it cannot be always due to pancreatic disease. Kaufman[32] has developed a new theory of diabetes. He shows that the pancreas exerts a restraining, and the liver an exciting, influence on the production of sugar. If the nerve-supply of either organ is cut off, puncture of the fourth ventricle still produces glycosuria, but if both are isolated, puncture produces no effect. Lately[33] he has shown that when the liver is isolated the effect of puncture on the pancreas is to cause only a temporary glycosuria by the shock.

Thus we are led to believe that the pancreas prevents glycosuria by an indirect action through the nervous system, or perhaps also by the direct action of its secretion on the hepatic cells. The close association of the two organs is shown by their embryogeny. The pancreas[34] is derived from three buds; two of these may develop into liver tissue in selachians, and in certain fishes the two organs remain permanently united and commingled. A brilliant discussion took place at Lyons, where Lancereaux, Lepine, and others discussed the cause of diabetes.[35] The first recognises three distinct diseases: pancreatic, nervous, and gouty glycosuria; and agrees with Kaufman that while the liver forms the sugar, the pancreas furnishes the means for its retention and transformation. Lepine holds that a soluble glycolytic ferment is imparted by the pancreas to the white blood cells, and probably other glands aid in this work. In diabetes there may be loss of this glycolytic power, or inability of the liver to form glycogen, or there may be too rapid change of glycogen into sugar. One or more of these factors may be present and the changes in the pancreas may be vaso-motor only. De Domenicis seems to be alone in asserting that even ligature of the pancreatic duct will produce diabetes, and that the so-called internal secretion is really poured into the intestine.[36]

The diagnoses of pancreatic from other forms of the disease may be aided by the observation of Lichtheim,[37] that besides the great emaciation there may have been previously attacks of epigastric pain vomiting and fever, with, later on, obstinate diarrhœa even without fatty stools. These attacks of pain were noted by Churton[38] in a case where a pancreatic cyst was incised and drained.

[29] Ed. Med. J., Dec. [30] Med. Rec., April 21st. [31] Lanc., April 27th. [32] Med Week, April 20th. [33] Med. Week, Nov. 2nd. [34] Med. Week., Sept. 28th [35] Med. Week, Nov. 2nd. [36] Med. Rec., April 31st. [37] Pract., April. [38] Lanc. June 2nd.

